# Assessment of the Psychometric Properties of the Danish VISA-P

**DOI:** 10.1155/2023/5291949

**Published:** 2023-05-02

**Authors:** Anne-Sofie Agergaard, Jonathan D. Comins, Volkert Siersma, Nikolaj M. Malmgaard-Clausen, Christian Couppe, Mikkel H. Hjortshoej, Jens L. Olesen, S. Peter Magnusson

**Affiliations:** ^1^Institute of Sports Medicine Copenhagen, Department of Orthopedic Surgery, Copenhagen University Hospital Bispebjerg and Frederiksberg, Denmark; ^2^Center for Healthy Aging, Department of Clinical Medicine, University of Copenhagen, Copenhagen, Denmark; ^3^Department of Physical and Occupational Therapy, Bispebjerg and Frederiksberg University Hospital, Copenhagen, Denmark; ^4^Section for Sports Traumatology M51, Bispebjerg and Frederiksberg University Hospital, Copenhagen, Denmark; ^5^Novo Nordisk A/S, Søborg, Denmark; ^6^The Research Unit for General Practice and Section of General Practice, Department of Public Health, University of Copenhagen, Copenhagen, Denmark; ^7^Research Unit for General Practice in Aalborg, Department of Clinical Medicine, Aalborg University, Aalborg, Denmark

## Abstract

**Purpose:**

The objective of the current study was to conduct a rigorous assessment of the psychometric properties of the Victorian Institute of Sports Assessment-patellar tendinopathy (VISA-P).

**Methods:**

Rasch analysis, confirmatory factor analysis (CFA), and multivariable linear regression were used to assess the psychometric properties of the VISA-P questionnaire in 184 Danish patients with patellar tendinopathy who had symptoms ranging from under 3 months to over 1 year. A group of 100 healthy Danish persons was included as a reference for known-group validation.

**Results:**

The analyses revealed that the 8-item VISA-P did not fit a unidimensional model, yielded at best a 3-factor model, and exhibited differential item functioning (DIF) across healthy subjects versus people with patellar tendinopathy.

**Conclusion:**

VISA-P in its present form does not satisfy a measurement model and is not a robust scale for measuring patellar tendinopathy. A new PROM for patellar tendinopathy should be developed and appropriately validated, and meanwhile, simple pain scoring (e.g., numeric rating scales) and functional tests are suggested as more appropriate outcome measures for studies of patellar tendinopathy.

## 1. Introduction

Patellar tendinopathy is a common injury that afflicts both elite and recreational athletes [1, 2] with an estimated prevalence as high as 45% in some sports [1]. Therefore, current efforts in ongoing research examine how to provide optimal treatment. The Victorian Institute of Sports Assessment-patellar tendinopathy questionnaire (VISA-P) [3] is the most widely used and the only condition-specific patient-reported outcome measure (PROM) for studies of patellar tendinopathy [4, 5]. A recently published consensus statement from the International Scientific Tendinopathy Symposium (ICON 2019) recommends the use of condition-specific PROMs such as VISA [6]. Moreover, PROMs are increasingly used as confirmatory outcomes in clinical trials and even prioritized over clinical and functional measures in the statistical testing hierarchy [7, 8]. Hence, to arrive at sound conclusions in clinical trials, a fundamental requirement is to confirm that the PROM exhibits adequate measurement properties for the targeted patient population and the specific trial conditions.

PROMs are important because a person's perception of a pathology, rather than the pathology itself, can be assessed [9]. Data derived from PROMs involve assigning numerical values to the response options to PROM questions (items). The (weighted or unweighted) scores from the individual items are then summed to an overall (total) score that, assuming unidimensionality, provides a proxy measure of the construct of interest [10]. Thus, in the case of the VISA-P, the lower the total score, the greater the perceived level of functional disability. Whether the PROM actually measures what it claims to measure depends on (1) the relevance and coverage of the items for the patient group being assessed (content validity) in relation to the construct of interest [11] and (2) whether the total response scores to those items satisfy the basic criteria of measurement (construct validity) [12–15]. When PROM data are congruent with (i.e., “fits”) statistical measurement models such as item response theory (IRT) models or confirmatory factor analysis (CFA) models, it follows that the PROM possesses adequate psychometric properties [16–18].

The VISA-P as a legacy PROMs was developed over 20 years ago. It has been reported to be a valid and reliable outcome measure for patients with patellar tendinopathy [3, 19]. However, these analyses were conducted only using classical test theory (CTT) methods, which today are considered insufficient to reach such a conclusion [20]. Since the original publication [3], VISA-P has been translated into several languages, including Danish, and has been reported to have acceptable cross-cultural validity [7, 21]. However, a recent thorough analysis found the content validity to be very poor [4]. The VISA-P was developed without input from patients with respect to the item generation and item reduction process [3]. Nor was there a description of the methodological background for PROMs development of the clinicians who developed the items or how and why they chose to include the eight items (and thus exclude other potential items). This process does not satisfy the general principles of establishing content validity, which requires face-to-face cognitive interviews with the targeted patients to confirm both the relevance, coverage, and comprehensibility of the items and response options [7, 14]. Furthermore, some items that possesses a mix of themes that addressing different domains and response options are problematic, and response option “yes” and “no” is likely a more appropriate response to some questions instead of the 11 possible response options on the 0–10 rating scale. Moreover, the measurement properties of VISA-P have never been assessed using robust statistical analytic methods [7].

The VISA-P has been used as an outcome measure in numerous clinical trials. Therefore, the aim of this study was to conduct a rigorous analysis of the psychometric properties of the Danish version of VISA-P in a cohort of patients with patellar tendinopathy and healthy controls.

## 2. Materials and Methods

The original version of the VISA-P questionnaire was published in 1998 [3]. It consists of 8 items with a maximum score of 100 for an asymptomatic, fully performing individual, and lower score indicating more symptoms and limitation of function and activity. Item 1 concerns the ability to sit without pain and the patient is asked to register the number of minutes of pain-free sitting (from 0-100 minutes) on a 0–10 numeric rating scale. Items 2–6 use the same 0–10 numeric rating scales as Item I, with 11 response options instead of adjective response scales. Item 7 has a 4-category response option structure, which is transformed to a 0–10 rating scale. The result is that items 1–7 can achieve up to 70 points, while item 8 has a maximum score of 30 points, and the VISA-P a maximum total score of 100. The original validation of VISA-P included only 38 patients and the calculation of a Pearson correlation between the VISA-P scores and a pain rating on the Nirschl pain scale [22].

In the present study the psychometric properties of VISA-P were assessed by looking at whether there was evidence of fit to an appropriate measurement model with a specific focus on evidence of unidimensionality and differential item functioning (DIF). Unidimensionality infers that responses to items depend only on a single characteristic (e.g. pain), which means that all items contribute to a single score [20]. DIF is a bias due to different response patterns in specific items between subgroups, such as sex, age group, or injury chronicity [23–26]. Evidence of DIF is detrimental to scale properties since it can mask real differences or detect differences between subgroups that are not attributable to real differences [25]. DIF is investigated by the use of models that assess the independence of a list of background variables on the items conditional on the summary VISA-P score. Unidimensionality is investigated by assessment of data fit to measurement models, such as confirmatory factor analysis (CFA) or item response theory (IRT) models [27, 28].

Rasch analysis, multivariable linear regression, and CFA were used to analyses the psychometric properties of VISA-P. The sample was a cohort of patients with patellar tendinopathy (symptom duration ranging from less than 3 months to more than a year) included in intervention studies at Bispebjerg Frederiksberg Hospital between 2006 and 2021, and a group of healthy persons. The study participants were male and females, 18 to 55 years of age, with clinical signs of patellar tendinopathy confirmed by ultrasound imaging. All patients were sports-active individuals (recreational or elite) recruited through the sports clinic at Bispebjerg and Frederiksberg Hospital, various local sports clubs, and online advertisement. Data were accessed from previous or ongoing trials at our facility: two studies included participants with symptoms <3 months: Tran et al. [29], ClinicalTrials.gov Identifier: NCT03642392 (ongoing), four studies included participants with symptoms >3 months: Kongsgaard et al. [30], Agergaard et al. [31], Olesen et al. [32], ClinicalTrials.gov Identifier: NCT04550013 (ongoing), and one study included both <3 month and >3 months: ClinicalTrials.gov Identifier: NCT04144946 (ongoing). All original studies were approved by the regional ethics committees, and all person identifiable data were removed prior to analyses.

The healthy controls were sports-active individuals of both sexes, 18 to 57 years old, recruited through local sports clubs and the University Copenhagen School of Sport Science, and who self-reported had no prior symptoms in their patellar tendons or previous knee surgery. Data were collected with no person identifiable data and therefore the participants only gave oral consent and permission from the regional ethics committees were not required in accordance with the ethical and scientific standards in Denmark.

## 3. Analysis Strategy

Multiple techniques were employed to assess the psychometric properties of VISA-P. First, fit to a Rasch unidimensional measurement model was assessed using Andersen's conditional likelihood ratio test (CLR). Overall fit was investigated through obtaining item-trait interaction chi-square values (a nonsignificant chi-square indicates good fit) [33, 34]. Individual item fit was assessed by standardized individual item-person fit residuals (i.e., the difference between observed and expected scores) to approximate a *Z*-Score, where values between ±2.5 indicates adequate fit to the model [33, 34]. As the item response structure of VISA-P consists of polytomous data, a partial credit polytomous model was applied. DIF was assessed using analysis of variance [35] for sex (male/female), age group (±30 yrs), BMI (±25), symptom duration (≤3 months, 4–12 months, ≥12 months, and no symptoms at all) [25, 36]. For age group DIF analyses, the cut-off of ±30 years of age was chosen because the median age of the sample group was 28 years. This allowed for dichotomization of younger versus older persons for comparison of scoring patterns across the groups. For BMI, the value of ±25 was chosen because this generally corresponds to the BMI cut-off value for being “overweight” [37]. The duration of symptoms groups were chosen to allow for a comparison of scoring patterns across groups with acute (<3 months), chronic (>3 months), and protracted chronic tendinopathy (>12 months).

The Rasch analysis for VISA-P was conducted analogously to a previous study of VISA for Achilles tendinopathy [38]. CFA was also used to assess four separate factor structures of the VISA-P: the original unidimensional structure, a reduced unidimensional scale including only item 2–6 (based on the results from the Rasch analysis), a 2-factor structure (items 1–5 and 6–8), and a 3-factor structure (items 1–3, 4–6, and 7-8). CFA model fit was assessed with the goodness of fit index (GFI) > 0.95; root mean square error of approximation (RMSEA) < 0.06; standardized root mean square residual (SRMR) < 0.06; and the comparative fit index (CFI) > 0.95 [39, 40].

Lastly, DIF was assessed for the same person characteristics as for the Rasch analyses (Sex, Age, BMI, and symptom duration) in multivariable regression analyses. These analyses were carried out for all subjects and repeated after removing the healthy subjects.

RUMM 2030 was used for the Rasch analysis [36]. CFA and regression analyses were carried out with SAS v9.4. SPSS AMOS v28 was also used for CFA and descriptive statistics.

## 4. Results

A total of 184 patients with patellar tendinopathy and 100 healthy participants were included in the present analysis.  Table 1 shows the characteristics of people in the sample, the variables used for the DIF analyses, and the VISA-P total scores across the subgroups.

### 4.1. Rasch Analysis

**Figure 1:**
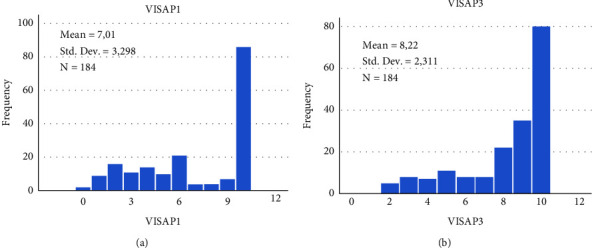
Response frequencies of symptomatic participants for item 1 (a) and 3 (b), showing substantial ceiling effect.

A ceiling effect was observed for certain items in VISA-P, notably for items 1 and 3.  Figure 1 shows the frequency distribution of response scores for items 1 and 3, which reveals a ceiling effect at the item level for patients with patellar tendinopathy, with over half the patients in the cohort essentially unable to improve in status on both these items.

The 0–10 response scales were recoded into 4 categories due to missing responses for all categories. The recoding structure was (0–2 = 0), (3–5 = 1), (6–8 = 2), and (9-10 = 3). Hence, a 4-category ordinal response scale was established for all items, which resulted in successful model estimation.  Table 2 shows that overall fit to the Rasch model was rejected for the combined item set (significant chi-square). Individually, items 2, 5, 6, and 7 exhibited misfit (Table 2). Incremental removal of item 7 followed by both items 7 and 8 did not remedy model misfit. Only after deleting items 1, 7, and 8 did a viable unidimensional model emerge.  Table 2 shows these results.

### 4.2. Confirmatory Factor Analysis (CFA)

Consistent with the Rasch results, the CFA rejected a unidimensional scale and indicated that a 3-factor structure with items 1–3, 4–6, and 7-8 in separate dimensions was more viable than the single factor 8-item scale (albeit not entirely convincing). The most robust CFA model confirmed the results from the Rasch analysis that showed that a reduced scale consisting of items 2–6 yielded the only plausible unidimensional scale for VISA-P.  Table 3 shows the CFA fit indices and Cronbach's alpha (*α*) for the different suggested scales.

For CFA and multivariable analyses, a cohort of healthy controls was included for assessment of known-groups validity and scaling properties across the spectrum of disease duration. The healthy control participants exhibited even greater ceiling responses at the item level than the symptomatic participants, and were only included in the CFA and multivariable analyses.

### 4.3. Multivariable Regression

Multivariable regression analyses showed considerable DIF in the original scale for all subjects (including both injured and healthy people), but the observed DIF was driven by the healthy subjects.  Table 4 shows the full DIF results. When the healthy subjects were removed from the analysis, DIF only remained for body mass index (BMI) in Item 8 (the asterisks in Table 4), which reveals that in the original 8-item form, VISA-P is not an adequate scale for comparison of healthy people with people with tendinopathy, or, importantly, for monitoring people with tendinopathy trying to become healthy.

## 5. Discussion

The main finding of the present study was that stringent psychometric assessment of the Danish version revealed that VISA-P does not satisfy a measurement model, lacks unidimensionality, and exhibits considerable DIF, which was driven by the healthy subjects. Furthermore, a reduced 5-item unidimensional VISA-P scale was supported by the psychometric assessment. Consequently, the use of VISA-P results can be misleading, as response to different treatments may be overlooked, or in worst case might lead to wrong conclusion in clinical trials.

The original validation of VISA-P included only 38 patients and the calculation of a Pearson correlation between the VISA-P scores and a pain rating on the Nirschl pain scale [22], but no assessment of the instrument's psychometric properties was undertaken. The present study included rigorous analyses of the psychometric properties in a broader sample of patients and healthy persons and revealed multiple problems with the VISA-P. Most importantly, it showed a lack of unidimensionality. Hence, when used as a single score in the original 8-item form, VISA-P is not an adequate scale for measuring self-reported impact of patellar tendinopathy.

Technical information regarding dimensionality was not provided in the original study [3]. Only one study has examined the factor structure using CFA in a sample of Spanish athletes [41], and they concluded a relatively acceptable fit of the one factor solution, although the study only assessed measurement invariance across sex, but not symptom duration, or response in a healthy population. Furthermore, they suggest that items 7 and 8 may not measure the same construct as the remaining 6 items. The present Rasch analysis shows substantial misfit for items 1, 7, and 8, which supports the viability of a reduced one-dimensional scale using only items 2–6, rather than a two-factor model. However, this might come at the price of incomplete content coverage, since physical activity and sports participation would be excluded. On the other hand, it would remove the heavily weighted item 7 and 8 from the total score, which results in an irrelevant score for athletes that continue training with symptoms or noncompetitive athletes without pain.

The VISA is not considered a diagnostic tool [3], however, there is a consensus among experts that the VISA can distinguish among healthy persons and those with tendinopathy, and that it is a good measure of symptom severity [42]. Importantly, the present evaluation demonstrated a violation of measurement invariance (DIF) across injured and healthy people, which renders the VISA-P an inadequate scale for comparison of healthy versus injured people, or to monitor injured people trying to recover in intervention studies. It would be more trustworthy to use simple pain scores or clinical tests (until a new PROM has been developed), allthough we do acknowledge that such measures cannot make up for a rigourosly developed PROM.

A recent COSMIN checklist review [43] found very low-quality evidence for the content validity for VISA-P. Furthermore, there is nothing to indicate that VISA-P was based on a theoretical reference model or patient involvement [3], which is necessary to confirm the relevance, coverage, and understandability of the items and response options of a PROM [7, 14]. Indeed, content validity is considered to be the most important measurement properties of a PROM [15] already questioning the suitability of the questionnaire. In addition, our psychometric evaluation of VISA-P using robust methods such as CFA or IRT also showed flawed construct validity. Therefore, the sufficient construct validity and responsiveness of the VISA-P found in the COSMIN review [44] based on the use of exploratory factor analysis and correlation with legacy instruments (criterion validity) cannot be confirmed. Hence, the results of the present study indicate inadequate measurement properties, and we suggest that the VISA-P should not be used or recommended for evaluation of patient with patellar tendinopathy. Moreover, it is paramount that researchers acknowledge the flaws of proposed measurement instruments, to avoid impeding further investigations to improve or develop better instruments. Until a new relevant condition-specific PROM for patellar tendinopathy has been developed, one can consider reanalyzing the existing data using a one-dimensional scale with only items 2–6, which was confirmed adequate in the present paper using CFA and Rasch analysis.

## 6. Conclusion

Rigorous psychometric assessment of the Danish version revealed that VISA-P does not satisfy a measurement model, lacks unidimensionality, and exhibits considerable DIF driven by the healthy subjects. A reduced 5-item unidimensional VISA-P scale was supported by the psychometric assessment, although content coverage remains unknown. A new relevant PROM for patellar tendinopathy should be developed and appropriately validated. Meanwhile, simple pain scoring (e.g., numeric rating scales) and functional tests are suggested as more appropriate outcome measures for studies of patellar tendinopathy.

## Figures and Tables

**Table 1:** Demographic variables for the DIF analyses and the total VISA-P scores.

		*N* (%)	Total score mean (SD)
Sex	Male	210 (73.9)	70.9 (19.9)
	Female	74 (26.1)	76.0 (21.8)

Duration (months)	≤3	64 (22.5)	64.9 (15.9)
	4–12	80 (28.2)	58.4 (14.7)
	>12	40 (14.1)	59.6 (17.4)
	No symptoms	100 (35.2)	92.9 (7.5)

Body mass index (BMI)	≤25	192 (67.6)	74.5 (21.0)
	>25	92 (32.4)	67.3 (18.5)

Age (years)	≤30	116 (40.8)	71.5 (21.0)
	>30	168 (59.2)	72.7 (20.1)

**Table 2:** Individual item fit and overall fit to the Rasch model.

	All items		Items 1–6 + 8		Items 1–6		Items 2–6	
Individual item fit								
	*χ* ^2^	*P*	*χ* ^2^	*P*	*χ* ^2^	*P*	*χ* ^2^	*P*
Item 1	6.085	0.048	10.218	0.006^*∗*^	14.064	0.0010^*∗*^		
Item 2	12.988	0.002^*∗*^	9.189	0.010	5.265	0.072	2.070	0.355
Item 3	5.235	0.073	5.415	0.100	1.388	0.500	1.293	0.524
Item 4	3.893	0.143	3.797	0.150	2.033	0.362	0.278	0.870
Item 5	11.034	0.004^*∗*^	5.833	0.054	4.983	0.083	0.916	0.633
Item 6	11.119	0.004^*∗*^	9.214	0.010	6.142	0.046	0.380	0.830
Item 7	26.322	<0.001^*∗*^						
Item 8	3.914	0.141	9.070	0.011				

*Overall fit*								
Total item *χ*^2^	80.590		52.738		33.874		4.937	
Degrees of freedom	16		14		12		10	
Total *χ*^2^ probability	<0.001^*∗*^		<0.001^*∗*^		<0.001^*∗*^		0.895	

*χ* ^2^ = Chi Sq, *P* = *p* value. ^*∗*^ = indicates a significant result at the 5% level.

**Table 3:** Results of the confirmatory factor analyses (CFA).

	GFI	RMSEA	SRMR	CFI	Cronbach's *α*
Target value	>0.95	<0.06	<0.06	>0.95	>0.70
1 dimension	0.931	0.1018	0.0695	0.8936	0.6482
1 dimension reduced	0.9914	<0.001	0.0200	1.0000	0.8174
3 dimensions	0.9506	0.0828	0.0496	0.9401	0.4430|0.7643|0.3336

GFI = goodness of fit; RMSEA = root mean square error of approximation; SRMR  =  standardized root mean residual; CFI  =  comparative fit index; 1 dimension = original scale item 1–8; 1 dimension reduced = only include item 2–6; 3 dimension = item separated in 1–3, 4–6, 7-8.

**Table 4:** Multivariable regression analysis for all subject of differential item functioning (DIF) on the covariates sex, duration of symptoms, body mass index (BMI), and age group.

	Item 1		Item 2		Item 3		Item 4		Item 5		Item 6		Item 7		Item 8	
	DIF (95% CI)	*p*-value	DIF (95% CI)	*p*-value	DIF (95% CI)	*p*-value	DIF (95% CI)	*p*-value	DIF (95% CI)	*p*-value	DIF (95% CI)	*p*-value	DIF (95% CI)	*p*-value	DIF (95% CI)	*p*-value
Sex		0.380		0.624		0.665		0.968		0.948		0.071		0.646		0.658
Male	Ref		Ref		ref		ref		ref		ref		ref		ref	
Female	−0.35 (−1.12; 0.43)	0.379	−0.10 (−0.52; 0.31)	0.624	−0.10 (−0.56; 0.36)	0.665	−0.01 (−0.48; 0.46)	0.968	0.02 (−0.49; 0.53)	0.948	0.42 (−0.04; 0.88)	0.071	−0.16 (−0.87; 0.54)	0.646	0.29 (−0.98; 1.56)	0.658

Duration (months)		0.915		0.002		0.158		0.179		0.005		0.142		<.0001		0.008
No Sympt	Ref		Ref		ref		ref		ref		ref		ref		ref	
<3	0.49 (−0.86; 1.84)	0.474	−1.07 (−1.79; −0.34)	0.004	−0.22 (−1.03; 0.58)	0.584	−0.53 (−1.35; 0.28)	0.202	1.35 (0.47; 2.24)	0.003	−0.19 (−0.99; 0.61)	0.647	3.81 (2.59; 5.04)	<.0001	−3.65 (−5.86; −1.45)	0.001
4–12	0.39 (−1.03; 1.81)	0.589	−1.20 (−1.97; −0.44)	0.002	0.42 (−0.43; 1.27)	0.331	−0.33 (−1.19; 0.53)	0.452	1.53 (0.59; 2.46)	0.001	0.47 (−0.38; 1.31)	0.279	2.64 (1.35; 3.93)	<.0001	−3.91 (−6.23; −1.58)	0.001
>12	0.34 (−1.13; 1.82)	0.649	−0.46 (−1.25; 0.34)	0.257	0.19 (−0.69; 1.07)	0.665	−0.87 (−1.76; 0.03)	0.058	0.81 (−0.16; 1.78)	0.102	0.26 (−0.61; 1.14)	0.556	3.20 (1.86; 4.54)	<.0001	−3.49 (−5.91; −1.07)	0.005

BMI (kg/m^2^)		0.459		0.267		0.769		0.176		0.003		0.734		0.783		^*∗*^
<25	Ref		Ref		ref		ref		ref		ref		ref		Ref	
>25	−0.27 (−0.98; 0.44)	0.459	−0.22 (−0.60; 0.17)	0.267	0.06 (−0.36; 0.49)	0.769	−0.30 (−0.73; 0.13)	0.175	−0.70 (−1.17; 0.23)	0.003	0.07 (−0.35; 0.49)	0.734	−0.09 (−0.73; 0.55)	0.783	1.43 (0.27; 2.59)	0.015

Age (years)		0.946		0.589		0.799		0.403		0.360		0.173		0.040		0.733
≤30	Ref		Ref		ref		ref		ref		ref		ref		ref	
≥30	0.02 (−0.62; 0.67)	0.946	0.10 (−0.25; 0.44)	0.589	0.05 (−0.33; 0.43)	0.799	0.17 (−0.22; 0.56)	0.402	0.20 (−0.23; 0.62)	0.359	0.27 (−0.12; 0.65)	0.172	−0.62 (−1.2; −0.03)	0.039	−1.18 (−1.24; 0.87)	0.733

The results show considerable DIF for all items. ^*∗*^indicate DIF remains (only for Item 8 for BMI) when healthy people are omitted from the analysis.

## Data Availability

The data used to support the findings of this study are available from the corresponding author upon request.
